# Predictive model of pheochromocytoma based on the imaging features of the adrenal tumours

**DOI:** 10.1038/s41598-022-06655-0

**Published:** 2022-02-17

**Authors:** Marta Araujo-Castro, Rogelio García Centeno, Cristina Robles Lázaro, Paola Parra Ramírez, Paola Gracia Gimeno, Patricia Martín Rojas-Marcos, Mariana Tomé Fernández-Ladreda, Juan Carlos Percovich Hualpa, Miguel Sampedro Núñez, María-Carmen López-García, Cristina Lamas, Cristina Álvarez Escolá, María Calatayud Gutiérrez, Concepción Blanco Carrera, Paz de Miguel Novoa, Nuria Valdés Gallego, Felicia Hanzu, Mónica Marazuela, Mireia Mora Porta, César Mínguez Ojeda, Isabel García Gómez Muriel, Héctor F. Escobar-Morreale, Pablo Valderrabano

**Affiliations:** 1 Department of Endocrinology & Nutrition, Hospital Universitario Ramón y Cajal, Instituto de Investigación Biomédica Ramón y Cajal (IRYCIS), Universidad de Alcalá, Madrid, Spain; 2 Department of Endocrinology & Nutrition, Hospital Universitario Gregorio Marañón, Madrid, Spain; 3 Department of Endocrinology & Nutrition, Hospital Universitario Virgen de La Concha, Zamora, Spain; 4 Department of Endocrinology & Nutrition, Hospital Universitario La Paz, Madrid, Spain; 5 Department of Endocrinology & Nutrition, Hospital Universitario Rollo Villanova, Zaragoza, Spain; 6 Department of Endocrinology & Nutrition, Hospital Universitario Puerto Real, Cádiz, Spain; 7 Department of Endocrinology & Nutrition, Hospital Universitario La Princesa, Madrid, Spain; 8 Department of Endocrinology & Nutrition, Complejo Hospitalario Universitario de Albacete, Albacete, Spain; 9 Department of Endocrinology & Nutrition, Hospital Universitario Doce de Octubre, Madrid, Spain; 10 Department of Endocrinology & Nutrition, Hospital Universitario Principe de Asturias, Madrid, Spain; 11 Department of Endocrinology & Nutrition, Hospital Universitario Clínico San Carlos, Madrid, Spain; 12 Department of Endocrinology & Nutrition, Hospital Universitario de Cabueñes, Asturias, Spain; 13 Department of Endocrinology & Nutrition, Hospital Clinic, Barcelona, Spain; 14 Department of Urology, Hospital Universitario Ramón y Cajal, Madrid, Spain; 15 Department of Diagnostic Imaging, Hospital Universitario Ramón y Cajal, Madrid, Spain

**Keywords:** Cancer imaging, Cancer screening, Endocrine cancer, Urological cancer

## Abstract

The purpose of our study was to develop a predictive model to rule out pheochromocytoma among adrenal tumours, based on unenhanced computed tomography (CT) and/or magnetic resonance imaging (MRI) features. We performed a retrospective multicentre study of 1131 patients presenting with adrenal lesions including 163 subjects with histological confirmation of pheochromocytoma (PHEO), and 968 patients showing no clinical suspicion of pheochromocytoma in whom plasma and/or urinary metanephrines and/or catecholamines were within reference ranges (non-PHEO). We found that tumour size was significantly larger in PHEO than non-PHEO lesions (44.3 ± 33.2 versus 20.6 ± 9.2 mm respectively; P < 0.001). Mean unenhanced CT attenuation was higher in PHEO (52.4 ± 43.1 versus 4.7 ± 17.9HU; P < 0.001). High lipid content in CT was more frequent among non-PHEO (83.6% versus 3.8% respectively; P < 0.001); and this feature alone had 83.6% sensitivity and 96.2% specificity to rule out pheochromocytoma with an area under the receiver operating characteristics curve (AUC-ROC) of 0.899. The combination of high lipid content and tumour size improved the diagnostic accuracy (AUC-ROC 0.961, sensitivity 88.1% and specificity 92.3%). The probability of having a pheochromocytoma was 0.1% for adrenal lesions smaller than 20 mm showing high lipid content in CT. Ninety percent of non-PHEO presented loss of signal in the “out of phase” MRI sequence compared to 39.0% of PHEO (P < 0.001), but the specificity of this feature for the diagnosis of non-PHEO lesions low. In conclusion, our study suggests that sparing biochemical screening for pheochromocytoma might be reasonable in patients with adrenal lesions smaller than 20 mm showing high lipid content in the CT scan, if there are no typical signs and symptoms of pheochromocytoma.

## Introduction

The increasing use of imaging techniques leads worldwide is driving an increase in the detection of adrenal incidentalomas (AIs), which are present in 4% of the general population, and in up to 10% of elderly patients^1^. After the diagnosis of an AI, its malignant nature and its hormonal production need to be assessed^2^. The diagnosis of adrenal cancer is usually established based on computed tomography (CT) and/or magnetic resonance imaging (MRI) studies due to the availability of highly specific radiological features^3^. However, a complex work-up is generally needed to assess its functionality. Hormonal evaluation must include the assessment of glucocorticoid excess in all cases; whereas mineralocorticoid and/or androgen excess are evaluated in selected patients based on clinical suspicion. Although pheochromocytomas are rare, current recommendations include ruling out catecholamine excess in all AIs to avoid the possibility of life-threatening crisis resulting from catecholamine excess^4^, by measuring urinary free metanephrines, urinary catecholamines and/or plasma free metanephrines^2^.

However, measurement of these hormones and metabolites is expensive, cumbersome, time consuming, and may be interfered by several drug and diet components often leading to falsely elevated results^5^. Moreover, although typical signs and symptoms of catecholamine excess are present in most patients with pheochromocytoma, up to 25% of them are asymptomatic and 50% present with only mild elevations of biochemical markers^6^. In this scenario, imaging plays a crucial role in differentiating cortical adenomas from pheochromocytomas. Even though no single imaging feature permits ruling out pheochromocytoma with confidence, earlier studies suggest that combinations of CT and/or MRI features are accurate enough as to avoid biochemical evaluation in some cases^7–10^. However, these studies have been typically conducted at single institutions with limited sample sizes, limiting the generalization of their results and their translation into clinical practice.

With this study we aimed to develop a predictive model based on imaging features of CT and or MRI studies which could reliably identify those adrenal tumours at very low risk of being a pheochromocytoma.

## Methods

This retrospective multicentre study was approved by the Hospital Universitario Ramón y Cajal and Hospital Universitario La Princesa Ethics’ Committees, and a waiver of informed consent was granted.

### Study population

We included a total of 1131 patients with adrenal lesions evaluated at 13 tertiary academic hospitals between 2001 and 2020 in whom imaging (CT and/or MRI) data were available.

**Figure 1:**
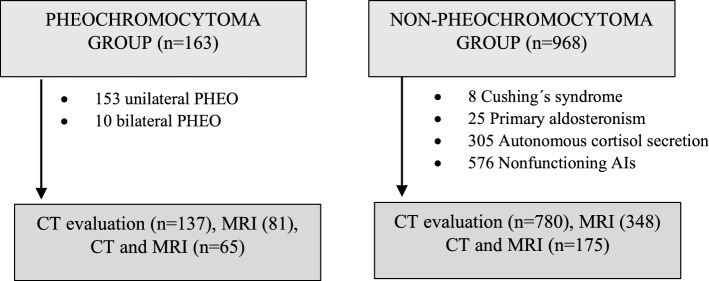
Study population. *AIs* adrenal incidentaloma, *CT* computed tomography, *MRI* magnetic resonance imaging. *In the PHEO group there were 85 patients with only CT available and 13 with only MRI; in the non-PHEO group there were 632 patients with only CT available and 161 with only MRI.

Patients were classified into two groups: (i) Patients with histological confirmation of pheochromocytoma (PHEO group) and (ii) Patients with urinary and/or plasma free metanephrines, and/or urinary catecholamine levels within reference range according to the different local laboratories and without clinical suspicion for pheochromocytoma (non-PHEO lesions). The latter were selected from a larger multicentre adrenal incidentaloma database, which included information on 968 patients presenting with one or more AIs of at least 1 cm in larger diameter and no catecholamine excess, evaluated at seven Spanish Hospitals between 2001 and 2020^11^. Patients in the first group were selected from the PHEO-RISK study database, which had information on 163 histologically confirmed pheochromocytomas who underwent adrenalectomy between 2005 and 2020 in ten Spanish tertiary hospitals^12^. Patients of both groups were identified through a systematic electronic search in the Pathology, Endocrinology, Biochemistry or Admission Departments files of the different hospitals (Fig. 1).

### Clinical and hormonal evaluation

Medical records were reviewed retrospectively to extract demographic information such as age, and sex, medical history of comorbidities at diagnosis including hypertension, type 2 diabetes mellitus, obesity, dyslipidaemia, cerebrovascular, and cardiovascular disease, and physical examination variables including body mass index (BMI) and systolic and diastolic blood pressure.

Hormonal evaluation consisted in at least the evaluation of catecholamine excess by the measurement of urinary (n = 588) or plasma free metanephrines (n = 32) or urinary catecholamines (n = 801) in all patients. In 496 patients, both metanephrine and catecholamine, were measured. Moreover, cortisol after dexamethasone suppression test (n = 905), plasma ACTH (n = 587), 24-urinary free cortisol (n = 441) and aldosterone/renin ratio (n = 638) were measured in some patients.

### Diagnostic imaging evaluation

All patients underwent unenhanced CT scan and/or MRI examinations at diagnosis (Fig. 1). Different equipment and image acquisition protocols were used throughout the study periods at different institutions. The following image features were extracted from study reports: (i) In CT studies, size (largest reported diameter), uni- or bilaterality, lipid content measured on unenhanced phase on the CT scan, presence of calcifications or necrosis, and Hounsfield units (HU); and (ii) in MRI studies: size (largest reported diameter) and *chemical shift imaging*, which allows the detection of intracellular lipid that is contained in most frequent adrenal lesions (adenomas) with loss of signal in the “out of phase” sequence^13^. For bilateral AIs, the size of the largest adenoma was included in the analyses. Adrenal tumours were considered rich in lipid content when attenuation was low (< 10 HU) in a CT performed without intravenous contrast^2^.

### Statistical analysis

Continuous variables were expressed as mean ± standard deviation and categorical variables were described as proportions. For variables with some missing data, we have indicated the number of patients with available results in brackets in the different tables. Shapiro Wilk’s test was used to assess normality of continuous variables and Levene’s test assessed homogeneity of the variances. Student’s t test was used for comparison of continuous variables, and χ^2^ test served for the comparison of proportions among the groups of patients. For quantitative variables reaching statistical significance in the comparisons, receiver operating characteristics curve (ROC) analysis was used as a measure of diagnostic accuracy, and to identify the cut-off values showing the best combination of sensitivity and specificity. The predictive model was developed using a multivariate logistic regression model. The selection of variables for the model was based on the results of the univariate logistic regression model to predict non-PHEO and only variables with less than 30% of missing results were considered to enter in the predictive model. The estimation of all possible equations was used to select the model with the best diagnostic accuracy (lower Akaike index (AIC) and maximum C Harrell index. ROC curve was also used to construct the model with the highest diagnostic accuracy. A two-tailed P value < 0.05 was considered as statistically significant in all analyses. All statistical data analyses were performed with STATA 15.0 (StataCorp LLC, College Station, Texas, USA).

### Ethical approval

All procedures performed in the participants of the study were in accordance with the ethical standards of the institutional research committee and with the 1964 Helsinki declaration and its later amendments or comparable ethical standards. The study has been approved by the Ethical Committee of the Hospital Universitario La Princesa and Hospital Ramón y Cajal University Hospital.

### Informed consent

This retrospective multicentre study was approved, and waiver of informed consent was granted by the Hospital Universitario Ramón y Cajal and Hospital La Princesa Ethics’ Committees.

## Results

### Patients

The comparison of the clinical characteristics of the 163 patients with PHEO with the 968 subjects with non-PHEO lesions is summarized in Table 1. Patients in the PHEO group were younger, leaner and had less frequently obesity and dyslipidaemia. Genetic information was available in 136 patients of the PHEO group, of whom 31.6% had a predisposing hereditary syndrome (27 MEN2A, 6 neurofibromatosis type 1, 4 SDHB mutations, 3 Von Hippel Lindau syndrome, 2 SDHD mutations and 1 patients MAX mutation). No differences were found in the prevalence of other cardiometabolic comorbidities.

**Table 1:** Baseline characteristics of the study population.

	PHEO (n = 163)	NON-PHEO (n = 968)	P value
Age (years)	51.7 ± 16.31	62.4 ± 11.13	< 0.0001^†^
Female sex	50% (n = 22)	39.0% (n = 16)	0.309
Hypertension	61.3% (n = 98)	54.0% (n = 522)	0.089
Type 2 diabetes	25.0% (n = 40)	24.7% (n = 238)	0.927
Dyslipidemia	34.6% (n = 55)	49.2% (n = 474)	0.001^†^
Cardiovascular events	13.8% (n = 22)	11.4% (n = 110)	0.384
Cerebrovascular events	4.4% (n = 7)	2.5% (n = 24)	0.177
Obesity	15.3% (n = 24)	37.7% (n = 306)	< 0.0001^†^
Systolic blood pressure (mmHg) (n = 913)	135.1 ± 18.23	135.1 ± 18.23	0.990
Diastolic blood pressure (mmHg) (n = 911)	80.3 ± 14.20	78.9 ± 10.90	0.269
Body mass index (kg/m^2^) (n = 784)	26.2 ± 5.33	29.4 ± 6.02	< 0.0001^†^

### Imaging and predictive model

The comparison of the imaging features of the PHEO and non-PHEO subgroups are summarized in Table 2. Among lesions evaluated with CT, mean tumour size was 20 mm larger in pheochromocytomas than in non-PHEO lesions, and the frequency of tumours above 40 mm was larger in the former. Calcification and necrosis were more common in pheochromocytomas, whereas high lipid content was much less frequent than in non-PHEO lesions. The unenhanced CT attenuation was higher in pheochromocytomas as was the frequency of lesions with attenuation > 10 HU. Bilaterality was more frequent in non-PHEO lesions. MRI showed a loss of signal in the “out of phase” sequence in 90.3% of the non-PHEO lesions compared with only 39% of pheochromocytomas. The typical hyperintensity in T2-weighted MRI studies was observed in 77.1% (64/83) of pheochromocytomas.

**Table 2:** Imaging features of PHEO and non-PHEO lesions.

	PHEO	non-PHEO	P value	OR [95% CI]
**Unenhanced CT evaluation**				
Tumour size (mm) (n = 857)	44.3 ± 33.2	20.6 ± 9.2	< 0.0001	1.12* [1.10–1.15]
Tumour size > 40 mm	44.9% (61/136)	2.6% (19/721)	< 0.0001	30.05 [17.04–53.00]
Hounsfield units (n = 136)	52.4 ± 43.07	4.7 ± 17.91	< 0.0001	1.07* [1.04–1.10]
Hounsfield units > 10 (n = 136)	94.9% (37/39)	20.6% (20/97)	< 0.0001	71.23 [15.81–320.97]
Bilaterality	6.3% (10/163)	23.8% (230/968)	< 0.0001	0.21 [0.11–0.40]
Necrosis (n = 873)	23.4% (26/111)	0.5% (4/762)	< 0.0001	1.23 [1.05–1.46]
Calcifications (n = 871)	5.5% (6/109)	1.4% (11/762)	0.004	3.97 [1.44–10.98]
High lipid content (n = 767)	3.8% (3/79)	83.6% (575/688)	< 0.0001	128.91 [39.96–415.84]
**MRI evaluation**				
Tumour size (mm) (n = 430)	38.3 ± 201.5	22.2 ± 10.00	< 0.0001	1.08* [1.06–1.11]
Loss of signal in the “out of phase” sequence (n = 390)	39.0% (23/59)	90.3% (299/331)	< 0.0001	0.07 [0.04–0.13]

The numbers in brackets make reference to n/N.

*For each increased in unit. Odds ratio (OR) were calculated by logistic regression analysis, being the reference group non-PHEO (non-PHEO = 0, PHEO = 1).

When using these features as single predictors of PHEO or non-PHEO lesions, HU showed the highest accuracy (91.7%) for PHEO lesions (AUC 0.917 [95% CI 0.866–0.968]), with a 16 HU threshold showing 89.7% sensitivity and 95.9% specificity, even though these measurements were not available in all patients. Accordingly, a low lipid content had 89.9% diagnostic accuracy for the prediction of PHEO lesions (AUC 0.899 [0.874–0.924]) with 89.7% sensitivity and 95.9% specificity for attenuation > 10 HU, whereas tumour size had 83.4% diagnostic accuracy (AUC 0.834 [95% CI 0.795–0.873]) for PHEO lesions with 76.6% sensitivity and 76.6 specificity for tumour size > 28 mm. On the contrary, the diagnostic accuracy of loss of signal in the “out of phase” sequence in MRI was only 75.5% accurate (AUC 0.757 [95% CI 0.692–0.8215]), with a 90.3% sensitivity but a 61.0% specificity for the presence of the loss of signal.

**Figure 2:**
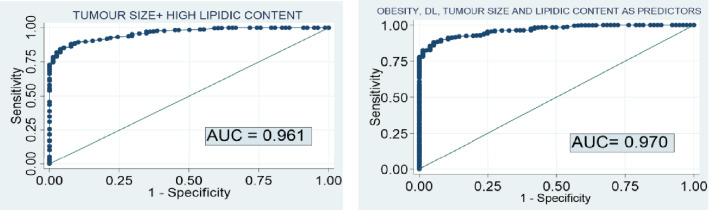
Diagnostic accuracy of the imaging features for the prediction of PHEO. AUC 0.961 [0.946–0.976]; Based on optimal threshold: Sensitivity 88.1%; Specificity 92.3%. AUC 0.970 [0.952–0.979]; Based on optimal threshold: Sensitivity 89.9%; Specificity 92.1%.

The combination of tumour size and high lipid content achieved a diagnostic accuracy of 96.1% for the diagnosis of non-pheochromocytoma (Fig. 2). Based on the predictive model, the probability of pheochromocytoma in an adrenal lesion smaller than 20 mm with high lipid content in CT scan was only 0.1% (Table 3). The diagnostic accuracy of the predictive model slightly increased when clinical variables (obesity and dyslipidaemia) were included in the model (Fig. 2).

**Table 3:**
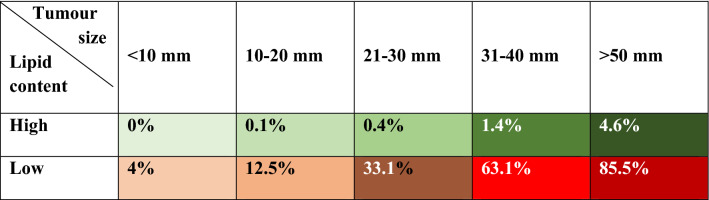
Probability of pheochromocytoma based on tumour size and lipid content.

The lowest probability of PHEO was observed in patients with adrenal lesions with a tumour size < 10 mm and high lipidic content (probability of PHEO = 0%), and the highest risk was seen in patients with adrenal lesions > 50 mm and low lipidic content (85.5%).

## Discussion

The predictive model developed in this study suggests that pheochromocytomas can be distinguished from other adrenal tumours with a high diagnostic accuracy based on the radiological features of unenhanced CT scan studies. A high lipid content is very specific for non-PHEO lesions (only 4% of pheochromocytomas in our series had high lipid content). Moreover, when high lipid content was combined with a small tumour size (< 20 mm), the probability that an adrenal lesion was a pheochromocytoma was below 0.1%.

In our series, pheochromocytomas were significantly larger than non-PHEO lesions and were frequently above 4 cm in diameter; in agreement with the findings of previous publications^14–17^. In this line, the mean tumour diameter in Gruber et al. metaanalysis was 38 ± 22 (range 12–150) mm; and approximately 40% of the tumours were larger than 4 cm in diameter^15^. We found that 28 mm was the tumour size threshold with the highest sensitivity and specificity for pheochromocytoma. Of note, a recent study found that tumours larger than 29 mm had a six-fold higher risk for being a pheochromocytoma than smaller lesions^14^.

A high lipid content based on unenhanced CT scan offered a specificity of 96.2% for the prediction of non-PHEO lesion in our cohort. It is known that most adenomas are rich in intracellular lipid content, leading to low attenuation values on unenhanced CT. In fact, attenuation values less than 10 HU are highly specific for adenomas^18^. However, 15 to 30% of adrenal adenomas show low lipid content^19^ making the differential diagnosis particularly challenging.

In our series, 16.4% of the non-PHEO lesions showed low-lipid content, whereas only 3 pheochromocytomas had high lipid content. Thus, a high lipid content can be considered very specific for non-PHEO lesions^20^. Accordingly, we found that HU were significantly higher in pheochromocytomas compared with non-PHEO lesions. A value above 16 HU showed 95.9% specificity for pheochromocytoma. Two previous meta-analyses found that a cut-off of more than 10 HU had a 100% sensitivity (95% CI, 1.00–1.00) for the diagnosis of pheochromocytoma^15,21^. For example, in the Gruber et al. metaanalysis, the mean unenhanced CT attenuation was 35 ± 9 HU, and only 15 tumours had attenuation ≤ 20 HU^15^. In this same line, Canu et al. states that it was calculated that 1232 patients harboring an adrenal tumor with an unenhanced attenuation value less than 10 HU needed to be biochemically screened to detect one pheochromocytoma^22^ as 0.5% of PHEOs had an attenuation of 10 HU. Moreover, in the Sane et al. series^17^ no patient with PHEO with an HU < 10, regardless of size, was described. We found that the combination of high lipid content with tumour size improved the diagnostic accuracy for pheochromocytomas in adrenal lesions. A similar observation had also been made in a previous smaller study^16^.

The chemical shift imaging in MRI is considered the best one to differentiate benign from malignant adrenal mass^3^.However, in our study the specificity of a loss of signal in the “out of phase” sequence of the MRI was too low to correctly identify non-PHEO lesions. Adrenal adenomas with high lipid content usually lose signal intensity on out-of-phase images compared with in-phase images, whereas malignant lesions and pheochromocytomas remain unchanged. However, in some cases, areas of fatty degeneration can be found, leading to slight signal drop on chemical shift^23^. Based on these findings, some studies recommend considering chemical shift as a second imaging test to further characterize a hyper-attenuating adrenal mass^24^. In this regard, MRI seems to be particularly useful to evaluate adrenal lesions with an unenhanced CT attenuation between 10 and 30 HU, while contrast-enhanced CT might be more useful for the evaluation of adrenal lesions with attenuation values above 30 HU^13^. Another typical finding of pheochromocytomas in MRI studies is the hyperintensity in T2-weighted images. We observed this finding in 77.1% of pheochromocytomas in our series, which is significantly higher than the 10% usually quoted in the literature^25^.

We must acknowledge some limitations of our study, starting by its retrospective design, which is prone to selection bias and missing data. Furthermore, radiological characteristics were extracted from imaging studies reports. As a consequence, we could not obtain precise HU measurements for many tumours, precluding us to include the exact HU units of the adrenal lesions in our predictive model. Also, the diagnosis of the non-PHEO lesions was mostly based on biochemical studies as most lacked histological confirmation because surgery was not appropriate for their management. Albeit it is possible that the non-PHEO group could include some non-secreting pheochromocytomas, this would be a rare event and thus, unlikely to change our findings. Moreover, imaging studies were acquired at different institutions with different equipment and image acquisition protocols. However, this supports the external validity of our current data, because this heterogeneity in equipment and image acquisition protocol characterizes daily clinical practice. Furthermore, the high consistency of our findings across different clinical sites suggests a robust diagnostic accuracy of radiological features for the discrimination of pheochromocytomas among adrenal lesions.

## Conclusions

Our study suggests that sparing biochemical screening for pheochromocytoma might be reasonable in patients with adrenal lesions smaller than 20 mm showing high lipid content in the CT scan, if there are no typical signs and symptoms of pheochromocytoma. For such adrenal lesions, the estimated probability of being a pheochromocytoma is below one in a thousand. However, further research is necessary to confirm our findings.
